# Neurology: Secondhand Behavioral Problems

**DOI:** 10.1289/ehp.115-a492a

**Published:** 2007-10

**Authors:** Carol Potera

Children whose mothers regularly smoked during pregnancy are known to be more likely to exhibit disruptive and aggressive behavior than children of nonsmoking mothers. A new study now finds that children born even to nonsmoking mothers who were exposed to chronic secondhand smoke (SHS) while pregnant face serious problems of attention deficit/hyperactivity disorder (ADHD) and conduct disorder, which includes fighting, truancy, substance abuse, and stealing.

At the University of Washington, psychologists Lisa Gatzke-Kopp and Theodore Beauchaine recruited 171 children aged 7–15 years who had emotional and behavioral problems. Their mothers consisted of 21 women who smoked regularly during the second and/or third trimester, 16 nonsmokers who were exposed during the second and/or third trimester to SHS at work or home, and 96 women who neither smoked nor were exposed to SHS (some of the mothers had more than one child in the study).

More symptoms of ADHD and conduct disorder were measured in children of smoking or SHS-exposed mothers, compared with children of smoke-free mothers. In fact, severe symptom scores were about the same for children whether they were exposed *in utero* to maternal smoking or to SHS. Childhood emotional disorders such as depression and anxiety were not significantly associated with maternal smoking or SHS exposure during pregnancy.

ADHD and conduct disorder are “externalizing” behaviors, as opposed to “internalizing” outcomes such as depression and anxiety. Animal models show that nicotine impairs brain development during the second and third trimesters, the formative periods for brain regions that control externalizing behaviors, such as the mesolimbic dopamine system.

The study, published online 23 May 2007 ahead of print in *Child Psychiatry and Human Development*, is the first to link SHS exposure in pregnancy to serious disruptive behavior during childhood. However, the level of SHS exposure studied was “chronic and extreme and all day long,” points out Gatzke-Kopp, now at Pennsylvania State University; therefore “pregnant women should not panic about minor exposures to secondhand smoke.” Still, the researchers hope their results will raise awareness about the potential health consequences of SHS to pregnant women in public places and work environments that still permit smoking (such as restaurants and bars).

Lauren Wakschlag, an associate professor of psychology at the University of Illinois Institute for Juvenile Research, says the new study “highlights where we need to go to disentangle the complex relationship of prenatal secondhand smoke exposure and antisocial behavior.” However, with only 16 SHS-exposed participants and 21 smokers, the central emphasis on SHS versus smoking needs to be interpreted cautiously. “These preliminary findings suggest a possibility that secondhand smoke affects behavior,” says Wakschlag, “but we need more systematic exploration of this.”

